# Quantifying and mapping species threat abatement opportunities to support national target setting

**DOI:** 10.1111/cobi.14046

**Published:** 2022-12-13

**Authors:** Louise Mair, Eduardo Amorim, Monira Bicalho, Thomas M. Brooks, Vincente Calfo, Renata de T. Capellão, Colin Clubbe, Marianne Evju, Eduardo P. Fernandez, Gláucia C. Ferreira, Frank Hawkins, Randall R. Jiménez, Lucas S. B. Jordão, Magni Olsen Kyrkjeeide, Nicholas B. W. Macfarlane, Bianca C. Mattos, Pablo H. A. de Melo, Lara M. Monteiro, Eimear Nic Lughadha, Nina Pougy, Domitilla C. Raimondo, Trine Hay Setsaas, Xiaoli Shen, Marinez Ferreira de Siqueira, Bernardo B. N. Strassburg, Philip J. K. McGowan

**Affiliations:** ^1^ School of Natural and Environmental Sciences Newcastle University Newcastle upon Tyne UK; ^2^ Instituto de Pesquisas Jardim Botânico do Rio de Janeiro Centro Nacional de Conservação da Flora Rio de Janeiro Brazil; ^3^ IUCN Gland Switzerland; ^4^ World Agroforestry Center (ICRAF) University of The Philippines Los Baños Laguna Philippines; ^5^ Institute for Marine & Antarctic Studies University of Tasmania Hobart Tasmania Australia; ^6^ International Institute for Sustainability Rio de Janeiro Brazil; ^7^ Royal Botanic Gardens Kew Richmond UK; ^8^ Norwegian Institute for Nature Research (NINA) Oslo Norway; ^9^ IUCN Washington, D.C. USA; ^10^ IUCN San José Costa Rica; ^11^ Norwegian Institute for Nature Research (NINA) Trondheim Norway; ^12^ WWF Portugal Lisbon Portugal; ^13^ South African National Biodiversity Institute Pretoria South Africa; ^14^ IUCN Species Survival Commission Pretoria South Africa; ^15^ State Key Laboratory of Vegetation and Environmental Change Institute of Botany Chinese Academy of Sciences Beijing China; ^16^ Rio Conservation and Sustainability Science Centre, Department of Geography and the Environment Pontifical Catholic University Rio de Janeiro Brazil

**Keywords:** habitat restoration, national red lists, species’ extinction risk, threat reduction, threatened species, vascular plants

## Abstract

The successful implementation of the Convention on Biological Diversity's post‐2020 Global Biodiversity Framework will rely on effective translation of targets from global to national level and increased engagement across diverse sectors of society. Species conservation targets require policy support measures that can be applied to a diversity of taxonomic groups, that link action targets to outcome goals, and that can be applied to both global and national data sets to account for national context, which the species threat abatement and restoration (STAR) metric does. To test the flexibility of STAR, we applied the metric to vascular plants listed on national red lists of Brazil, Norway, and South Africa. The STAR metric uses data on species’ extinction risk, distributions, and threats, which we obtained from national red lists to quantify the contribution that threat abatement and habitat restoration activities could make to reducing species’ extinction risk. Across all 3 countries, the greatest opportunity for reducing plant species’ extinction risk was from abating threats from agricultural activities, which could reduce species’ extinction risk by 54% in Norway, 36% in South Africa, and 29% in Brazil. Species extinction risk could be reduced by a further 21% in South Africa by abating threats from invasive species and by 21% in Brazil by abating threats from urban expansion. Even with different approaches to red‐listing among countries, the STAR metric yielded informative results that identified where the greatest conservation gains could be made for species through threat‐abatement and restoration activities. Quantifiably linking local taxonomic coverage and data collection to global processes with STAR would allow national target setting to align with global targets and enable state and nonstate actors to measure and report on their potential contributions to species conservation.

## INTRODUCTION

The post‐2020 Global Biodiversity Framework (GBF) is developed and agreed on at the global level by the Parties to the Convention on Biological Diversity (CBD) (CBD, 2021). The framework and associated targets provide the conservation policy agenda globally. However, conservation action is taken at the national level, so directly linking the 2 is critical for successful implementation. An important aspect of the failure to implement the CBD's preceding Aichi Biodiversity Targets (part of the Strategic Plan for Biodiversity 2011–2020 [CBD, 2010]) was the weak translation from global targets to national targets (Xu et al., 2021); most national targets are poorly aligned to Aichi Targets (CBD Secretariat, 2020). This, exacerbated by a lack of engagement of nonstate actors in the strategic plan (Smith et al., 2020), resulted in a failure to integrate biodiversity in decision‐making across policy, business, and society. It is therefore essential to achieving transformative change that post‐2020 targets are effectively translated to the national level, while accounting for national contexts (Xu et al., 2021), and that all sectors of society are engaged in biodiversity conservation (Milner‐Gulland et al., 2021; Smith et al., 2020).

A recently developed measure that can support implementation of the GBF at national levels is the species threat abatement and restoration (STAR) metric (Mair et al., 2021), which was developed to support science‐based target setting for species conservation across spatial scales. The STAR metric utilizes red‐list data on species’ extinction risk and documented threats to species and species’ current area of habitat (AOH) data and restorable AOH (natural habitat that has been lost and could be restored) to quantify the potential contribution of threat abatement and habitat restoration activities in a particular place to reduction of global species extinction risk (Mair et al., 2021). The metric defines, for each species, a global STAR threat‐abatement score (STAR*_T_*). This ranges from 0 for least‐concern species to 400 for critically endangered species. The sum of STAR*_T_* across all species represents the global threat abatement effort needed, in principle, for all species to become least concern. This total threat‐abatement score can be disaggregated by threat with red‐list data on the relative contribution of threats to species’ extinction risk and spatially with the species’ current AOH. The STAR restoration score is calculated using the extent of species’ restorable AOH relative to current AOH and is based on the assumption that threat‐abatement activities are required in restorable AOH.

The STAR metric can thus be used to quantify the potential contribution of actions taken in response to the GBF action targets (targets 1–8 focus on reducing threats to biodiversity and restoring ecosystems) toward the achievement of the outcome goals and milestones (goal A focuses on the state of biodiversity) (CBD, 2021). A real strength of STAR is that it enables science‐based targets to be set for any given site of any size. The metric therefore has the potential to allow not only governments, but also nonstate actors, such as nongovernmental organizations, civil society, and business, to engage in the GBF by making and relating their own voluntary commitments to species conservation with quantifiable outcomes.

To date, the STAR metric has been applied to terrestrial amphibian, bird, and mammal species listed as globally threatened (vulnerable, endangered, or critically endangered) or near threatened on the International Union for Conservation of Nature (IUCN) Red List of Threatened Species (Mair et al., 2021). The metric has the potential to be applied to other data sets and taxa because it requires, at a minimum, documentation of species’ extinction risk, distribution, and threats faced. Specifically, national red lists hold great potential for supporting further application of the STAR metric. The establishment of national red lists has been a primary focus of Parties to the CBD in response to the Aichi Targets (CBD, 2016), and in many countries such lists help shape national policy and conservation planning (e.g., Raimondo, 2017). The extension of STAR to incorporate national red‐list data therefore has the potential to strengthen implementation of the GBF by providing a tool that is further tailored to the national context. National red lists also provide expanded taxonomic scope compared with the global IUCN Red List. For example, although >30% of plant species have a global extinction risk assessment (Nic Lughadha et al., 2020), just 13% of plants are represented by assessments on the IUCN Red List (IUCN, 2021) because most assessments of plant conservation status are published as part of national or regional red‐list initiatives (Mounce et al., 2018).

There is great value in developing metrics that link action targets to outcome goals and are applicable to a diversity of taxonomic groups and to global and national data sets. The STAR metric embodies these attributes. We applied the STAR metric to national red lists for vascular plants from 3 contrasting countries: Brazil, Norway, and South Africa. Brazil is home to more native vascular plant species than any other country in the world, 55% of which are national endemic species (Brazil Flora Group, 2022), and has placed a strong emphasis on developing extinction risk assessments (Martins et al., 2017). The extinction risk of 22% (7830 out of 35,683) of terrestrial plant species has been assessed by Brazil's National Center for Plant Conservation (Centro Nacional de Conservação da Flora, CNCFlora). South Africa holds 5% of the world's plant diversity (20,456 species) and was the first megadiverse country to develop a comprehensive national red list of plant species (Raimondo et al., 2013). Norway is a high‐latitude country, where many species reach the northern‐ or southern‐most limit of their distributions, and the country has comprehensive red list assessments for many less well‐studied taxa, including all plant species (Henriksen & Hilmo, 2015b).

Applying STAR to plant data for the first time, our aim was to understand the opportunities and limitations of application of the STAR metric to national red lists in different national contexts, where different types of data are available, and to determine what STAR can reveal about the opportunities for threat abatement to reduce the extinction risk of plant species in these 3 countries. Red list assessment protocols and data availability vary among countries, and so we adapted the STAR approach to suit each case, applying the threat‐abatement and restoration components of STAR to the South Africa study, but only the threat‐abatement component to the Norway and Brazil studies. We considered our results in the context of national policy and the GBF.

## METHODS

### STAR metric

The STAR metric data requirements, calculation, and theoretical basis are presented in detail in Mair et al. (2021). In brief, the global application of STAR makes use of global IUCN Red List data. Species’ extinction risk is categorized on the IUCN Red List based on species’ population or distribution data or both (Mace et al., 2008). As part of the red‐listing process, species experts document the known threats to species based on a hierarchical threat classification scheme (e.g., the specific classification “1.1 housing & urban areas” is nested within the general classification “1 residential & commercial development” [Salafsky et al., 2008]). The scope (proportion of total population affected) (e.g., “affects the minority of the population [<50%]”), severity (overall declines caused by the threat) (e.g., “causing or likely to cause very rapid declines [>30% over 10 years or 3 generations; whichever is the longer]”), and timing (e.g., “ongoing”) of threats are also documented where possible.

The STAR threat‐abatement score (*T*) for a location (*i*) and threat (*t*) is calculated among all species as

(1) $$T_{t,i}=\sum_{s}^{N_{s}}P_{s,i}W_{s}C_{s,t},$$

where *P_s,i_* is the current AOH (Brooks et al., 2019) of each species (*s*) in location (*i*) expressed as a percentage of the global species’ current AOH; *W_s_* is the IUCN Red List category weight of species *s* (1, near threatened; 2, vulnerable; 3, endangered; 4, critically endangered); *C* is the relative contribution of threat *t* to the extinction risk of species *s*; and *N_s_* is the total number of species at location (*i*). The relative contribution of each threat (following a standard threats classification scheme [Salafsky et al., 2008]) to the species’ extinction risk was calculated as the percent population decline from that threat (derived from the product of severity and scope for that threat in each species’ IUCN Red List assessment) divided by the sum of percent population declines from all threats to that species. For an example calculation of the STAR threat‐abatement score, see Appendix S1. Scope and severity data are often missing from red list assessments; however, earlier sensitivity analyses that replaced varying proportions of documented scope and severity scores with the median of possible scores showed that overall results remain similar, particularly at sites with large numbers of species (Mair et al., 2021). Thus, using the median of possible values of scope and severity to replace missing data was considered a suitable approach.

The STAR restoration component is not based on assumptions about the extent of habitat restoration required for individual species; instead, it quantifies the potential contribution habitat restoration activities could make to reducing species’ extinction risk (Mair et al., 2021).

The STAR restoration score (*R*) for the potential contribution of habitat restoration (and threat abatement therein) at location *I* for threat *t* is calculated as

(2) $$R_{t,i}=\sum_{s}^{N_{s}}H_{s,i}W_{s}C_{s,t}M_{s,i},$$

where *H_s,i_* is the extent of restorable AOH for species *s* at location *i* expressed as a percentage of the global species’ current AOH and *M_i_* is a multiplier appropriate to the habitat at location *i* to discount restoration scores. We used a global multiplier of 0.29 based on the median rate of recovery from a global meta‐analysis (Jones et al., 2018) under the assumption that restoration has been underway for 10 years. The global multiplier provides an initial estimate of the potential contribution of habitat restoration to species’ extinction risk reduction and should be refined based on local restoration potential for site‐specific applications of STAR.

For each species, the total STAR*_T_* score could be achieved by the complete abatement of all threats in remaining habitat or an equivalent value of the STAR metric can be achieved by a combination of threat abatement in remaining habitat and restoration of lost habitat (with concomitant threat abatement therein).

The STAR metric was adapted to each case study country context as necessary to facilitate the use of national red list data. The specific methods applied to each country are detailed below and differences are summarized in Table 1. All analyses and data visualization were carried out in R (R Core Development Team, 2018).

**TABLE 1:** The methodological adaptations made in the application of the species threat abatement and restoration (STAR) metric to vascular plants in 3 countries and the associated justifications and assumptions made

	Country		
STAR component	Brazil	Norway	South Africa
Species included	Approach and adaptation		
	Threatened (VU, EN, and CR) and near‐threatened (NT) endemic species that have been assessed and have necessary data; ∼22% of Brazilian terrestrial flora have been assessed	All VU, EN, and CR and NT species (endemic and nonendemic) with required data; Norwegian vascular plants have been comprehensively assessed	All VU, EN and CR endemic species for which required data were available; South African threatened endemic species have been comprehensively assessed
	Justification and assumptions		
	Sample has some biases (see text), but it includes assessed taxa across all of Brazil's 6 major biomes; restriction of analyses to endemic species means STAR scores unambiguously reflect potential contributions to global goals, but underestimates entire potential contributions	Inclusion of endemic and nonendemic species captures full potential contribution to species’ extinction risk; national extinction risk category of nonendemic species not necessarily the same as the global extinction risk category (see weight of species below)	Range maps not available for NT species; their exclusion underestimates total STAR score for South African endemic plants; restriction of analyses to endemic species means STAR scores unambiguously reflect potential contributions to global goals, but underestimates entire potential contributions
Threat abatement			
Weight of species (derived from red‐list category)	Approach and adaptation		
	Same as global approach because only endemic species included	Nonendemic species assessed according to their national (not global) distribution, resulting in national extinction risk categories (and therefore weight of species in calculation) that differed from species’ global extinction risk categories	Same as global approach because only endemic species included
	Justification and assumptions		
		Inclusion of nonendemic species assessed according to their national (and not global) distribution means; STAR results are specific to the national context (but see “current area of habitat” below for additional refinement).	
Current area of habitat (AOH)	Approach and adaptation		
	Species’ extent of occurrence (EOO) polygons used to map species’ extent without refinement to species’ habitat associations	Estimated share of species’ global population used to weight nonendemic species; occurrence of species per county used to map STAR scores; county area used to weight relative distribution of species among counties	Species range polygons (already clipped to suitable vegetation type and elevational range) overlaid with land‐cover maps at 30‐m resolution; cells with natural habitat in both periods (1990 and 2014) (Appendix S5) used to map current AOH, which was aggregated to 300‐m resolution
	Justification and assumptions		
	Species extent overestimated compared with their AOH; EOO polygons affected by spatial variation in sampling effort	Species extent overestimated compared with their AOH; species range maps do not exist for all nationally red‐listed species	Species current AOH mapped as accurately as possible and is an improvement in terms of accuracy and spatial resolution compared with the global methods applied in Mair et al. (2021)
Relative contribution of threat to extinction risk (derived using threat scope and severity)	Approach and adaptation		
	Threat classification: proportion of assessments carried out with a country‐specific threat classification scheme; these were translated to IUCN threat classification scheme (Appendix S2)	Threat classification: specific to the country (Appendix S3)	Threat classification: as in the global application, IUCN threat classification system used
		Scope and severity: as in the global study; median values used to replace missing scope and severity scores	
			Scope and severity: as in global study; median values used to replace missing scope and severity scores
	Scope and severity: scoring approaches inconsistently applied during assessments, so all threats weighted equally		
	Justification and assumptions		
	Threat classification: translation allowed all available assessments to be used in the STAR analysis; result was some loss of threat detail (e.g., translation of threats to the coarse category natural system modification)	Threat classification: threat categories not comparable to global threat categories; most relevant to the national context	
	Scope and severity: all threats assumed to have same impact on species		
Restoration			
Restorable area of habitat	Approach and adaptation		
	Not applied	Not applied	Species range polygons overlaid with land‐cover maps; cells with habitat types considered potentially restorable (see METHODS and Appendix S5) used to map restorable AOH, which was aggregated to 300‐m resolution
	Justification and assumptions		
			Approach is an improvement on global application because it identifies restorable AOH as the habitat types most likely to be suitable for restoration

Abbreviations: AOH, area of habitat; CR, critically endangered; EN, endangered; EOO, extent of occurrence; IUCN, International Union for Conservation of Nature; NT, near threatened; VU, vulnerable.

### Brazil

Data on the national red list status of plants in Brazil were obtained from CNCFlora (CNCFlora/JBRJ, 2021). These data are from national red list assessments conducted from 2010 to 2020 and include species’ extinction risk category, documented threats to species, and extent of occurrence (EOO) maps. The CNCFlora has fully assessed the extinction risk of nearly 22% (7830 out of 35,683 species) of the Brazilian terrestrial flora, of which 41% (3213 species) were evaluated as threatened and nearly 6000 are national endemic species. Although this sample has selection (e.g., species previously assessed as threatened during other risk evaluation processes [Martinelli & Moraes, 2013] and trees under the Global Tree Assessment initiative [globaltreeassessment.org]) and geographic biases (confined to certain species‐rich areas of Brazil, e.g., Rio de Janeiro state endemic species [Martinelli et al., 2018]), represented taxa nevertheless have been assessed across all of Brazil's 6 major biomes, in the biodiversity hotspots (Atlantic Forest and Cerrado) and in remote, poorly known regions across the country.

Because CNCFlora focuses on endemic species, we included only endemic plant species assessed as near threatened and threatened (vulnerable, endangered, or critically endangered) that were taxonomically accepted in Flora do Brasil (2020). Although the exclusion of nonendemic species means that the resulting STAR scores do not represent the entire potential contributions toward global goals, restricting the analysis to endemic species had the advantage that species’ extinction risk (and hence resulting STAR scores) were the same at the national and global scales. In contrast, the national extinction risks of nonendemic species may vary among countries and differ from the species’ global extinction risk (Brito et al., 2010). Assessments of taxa below the species level were excluded because these have not been assessed comprehensively. Where species had more than 1 assessment in the CNCFlora database, the most recent assessment was used.

Assessments carried out before 2013 followed IUCN (2012), but the threat classification system was specific to Brazil, which was adapted from a threat classification system previously proposed by IUCN. More recent assessments used the updated IUCN global threat classification scheme (Salafsky et al., 2008). To allow data from all of these assessments to be used in the STAR analysis, we developed a key with which we could match as closely as possible the Brazilian threat classes to the IUCN threat classes and translated all threats accordingly (Appendix S2). Threats were recorded at different levels in the classification hierarchy, depending on the level of information available for individual species assessments. Threat timing was not recorded and so was not used. Approaches to scope and severity scoring were not consistent during the assessment process and so were not used. Instead, we assumed all threats had equal scope and severity.

We calculated STAR*_T_* scores with Equation (1). The relative contribution of each threat to the species’ extinction risk was calculated simply as 1 divided by the number of threats per species (i.e., all threats were weighted equally) (for justification of this approach, see sensitivity analyses in Mair et al. [2021]).

The total threat‐abatement score was mapped at 25‐km resolution with the species’ EOO polygons. Each species EOO polygon was converted to a presence–absence raster (recognizing that this will generate substantial commission errors [Rondinini et al., 2006]), and the STAR*_T_* score per grid cell was calculated by multiplying each species’ total STAR*_T_* score by the proportion that the grid cell represented of the total species grid cells. A map of total STAR*_T_* scores was produced by summing the STAR*_T_* score maps across all species. Habitat classification and elevation data were not consistently recorded during species assessments, which are required data to calculate species AOH (e.g., see “South Africa” below), so we were unable to map current or restorable AOH or calculate STAR*_R_*.

### Norway

The 2015 Norway national red list was used (Henriksen & Hilmo, 2015a), which followed IUCN (2012) and is comprehensive for vascular plant species. We included species assessed as near threatened or threatened. The global IUCN criteria do not include a quantitative threshold for near‐threatened species; however, the Norwegian Biodiversity Information Center has set thresholds for use in Norwegian red list assessments. We included only species that occurred on mainland Norway because species were assessed separately for the Norwegian islands of Svalbard, resulting in some species that occur in both having 2 different extinction risk assessments (Henriksen & Hilmo, 2015b). Assessments of taxa below the species level were also excluded.

Threats to species were documented according to a hierarchical classification scheme specific to the Norwegian national red list (Henriksen & Hilmo, 2015c; Ødegaard et al., 2005), and we translated threat names into English (Appendix S3). Threat timing, scope, and severity were documented. The Norwegian red list's version of the threat severity categories is simplified; there are only 3 categories (“causing or likely to cause rapid declines,” “causing or likely to cause relative slow but significant declines,” and “insignificant/no declines” [Norwegian Biodiversity Information Center, 2014]). As in Mair et al. (2021), threats documented as “past and unlikely to return” were excluded. Where scope or severity data were missing or unknown (15% of cases for scope and 38% for severity), median values were used (i.e., we assumed scope was the majority of the population [50–90%] and severity was causing slow but significant declines [<20% over 10 years or 3 generations], as in Mair et al. [2021]).

We calculated STAR*_T_* scores with Equation (1). Given that species included were endemic and nonendemic species, we used the estimated share of each species’ global distribution occurring in Norway (documented during red‐list assessments) to inform *P_s,I_* (percentage of the distribution of each species *s* in location *i*). Estimated global shares were given as percentage bands (with endemic species documented in the >50% share band), and we used the minimum, median, and maximum estimated share in calculations to include a measure of uncertainty (Appendix S4).

The total threat‐abatement score was mapped at the spatial resolution of countries, reflecting the availability of species distribution data. Assessments classified the occurrence of species at the county level as certain, uncertain, or absent; we included only certain occurrences. We made the simplifying assumption that species occurred at the same density within each county and used the area of each county (from 2225 km^2^ for Vestfold to 48,631 km^2^ for Finnmark; mean county area = 20,279 km^2^) that species occurred in to calculate the proportion of species’ national distribution within each county. We also used the median estimated share of the species global population; thus. *P_s,i_* (where *i* is county) was informed jointly by the estimated share of species global population and the estimated proportion of species national distribution per county. We did not calculate STAR*_R_* because the species distribution data did not allow for AOH (either current or restorable) to be mapped.

### South Africa

The South Africa national red list of plants 2020 was used (SANBI, 2020). Species are assessed following IUCN (2012). The list is comprehensive for vascular plant species in South Africa. Given that 67% (13,763) of South Africa's 20,401 plant species are endemic (Skowno et al., 2019), we considered only endemic species, which means the calculated STAR scores reflect only partial potential contributions toward global goals. We included only species assessed as threatened. Near‐threatened species were excluded because their distributions have not been mapped.

Assessments documented threats to species (Salafsky et al., 2008), threat timing, scope, and severity. The only deviation from the IUCN Red List methodology is that the severity categories “no declines” and “causing or could cause fluctuations” were not used (these categories have not been implemented in the South African red‐listing process). As in Mair et al. (2021), threats documented as “past and unlikely to return” were excluded. Where scope or severity data were missing or unknown (45% of cases for scope and 18% for severity), median values were used (i.e., scope was assumed to be the majority of the population [50–90%] and severity was assumed to be causing slow but significant declines [<20% over 10 years or 3 generations], as in Mair et al. [2021]).

Threatened plant species’ distribution data were also available and were used to develop species’ current AOH and restorable AOH. To determine the distribution of current natural habitat and potentially restorable habitat, we used a nationally generated land‐cover map that captured both the land‐cover type in 2014 and the change in land cover since 1990 at 30‐m resolution (provided by the South African National Biodiversity Institute and following methods in Skowno et al. [2021]). Current habitat was identified as cells that contained natural habitat classes in both periods (Appendix S5). Restorable habitat was identified as cells that: were secondary natural habitat and contained erosion; were converted from natural habitat to cropland, plantations, or mines from 1990 to 2014; had been eroded from 1990 to 2014; or were mines in both periods (Appendix S5). All other cells were considered not natural and not restorable and were therefore excluded from current and restorable habitat (Appendix S5). Binary maps of current and restorable habitat were produced at 30‐m resolution and were aggregated to give proportion of habitat per grid cell at 300‐m resolution (Appendix S6). To calculate species current AOH, the proportion of current natural habitat per 300‐m grid cell was overlaid with the species distribution maps. For restorable AOH, the proportion of restorable habitat per 300‐m grid cell was overlaid with the same species distribution maps. Species restorable AOH therefore reflected the potentially restorable habitat within the species’ current distribution extent. Data on species historical distributions were not available, and this approach provided conservative estimates of species restorable AOH.

We used Equations (1) and (2), respectively, to calculate STAR*_T_* and STAR*_R_*. We used species current AOH and restorable AOH, respectively, to map STAR*_T_* and STAR*_R_*. For each species, the STAR*_T_* score per grid cell was calculated by multiplying each species’ total STAR*_T_* score by the proportion of the species’ current AOH in the grid cell. The STAR*_R_* score per grid cell was calculated by multiplying the species’ total STAR*_R_* score by the proportion of species’ restorable AOH present in the grid cell. Maps of total STAR*_T_* and STAR*_R_* scores were produced by summing the respective score maps across all species.

## RESULTS

### Brazil

The total number of threatened and near‐threatened endemic plant species in Brazil based on data suitable for inclusion was 2791 (172 near threatened, 541 vulnerable, 1492 endangered, and 586 critically endangered).

**FIGURE 1:**
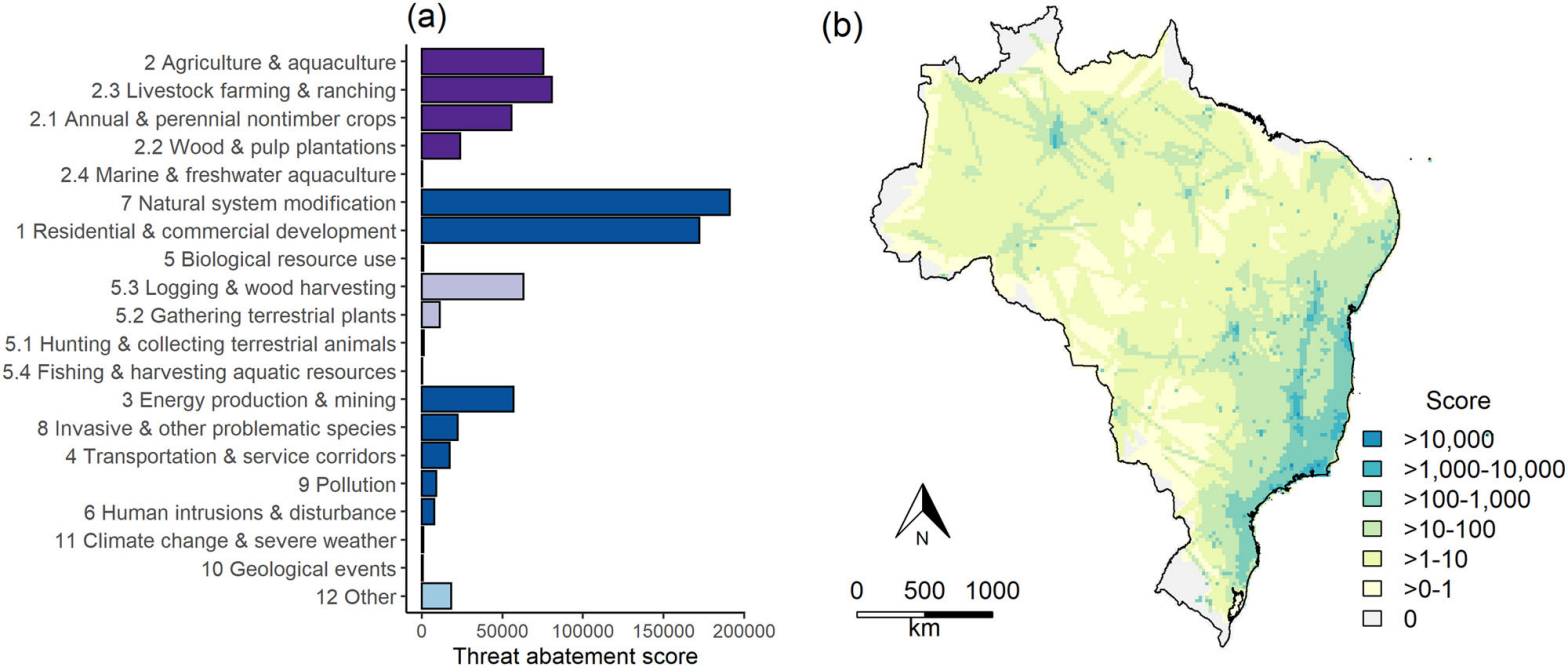
For threatened endemic species of plants in Brazil, (a) threat‐abatement score for each threat category (threat classification numbers and names according to the International Union for Conservation of Nature Red List threat classification system; dark purple, threats from agriculture; light purple, threats from biological resource use; blue, threat category other; dark blue, remaining threats) and (b) total threat‐abatement scores mapped at the 25‐km resolution

The total STAR*_T_* score for plant species in Brazil for which assessments were available was 807,400. Threats from agriculture made a larger contribution to the total STAR*_T_* score (29%) than any other category of threat, with 9% from general agriculture (classified at the first level in the threat classification hierarchy), 10% from livestock farming and ranching, 7% from annual and perennial nontimber crops, and 3% from wood and pulp plantations (Figure 1a). Following this, natural system modification contributed 24% (with changes in fire frequency and intensity contributing 10%, dams and water management contributing 1%, and other ecosystem modifications contributing 13%, which is an artifact of the translation of the Brazilian threat classes to the IUCN threat classes [Appendix S2]) and residential and commercial development contributed 21% to the total STAR*_T_* score.

The mapped STAR*_T_* scores in Figure 1b reflect the accumulating threats in the Atlantic Forest and Cerrado (Brazilian savanna) biodiversity hotspots, as well as the impact of national roads in areas such as the Amazon, presumably both as a vector of pressure and in that they allow access for botanical survey effort (Oliveira et al., 2016).

### Norway

All 1357 species of vascular plants native to Norway have been assessed on the national red list. Of these, 301 species were assessed as near threatened or threatened and had documented threats and distribution information available and were therefore included in this analysis (104 near threatened, 87 vulnerable, 87 endangered, and 23 critically endangered).

**FIGURE 2:**
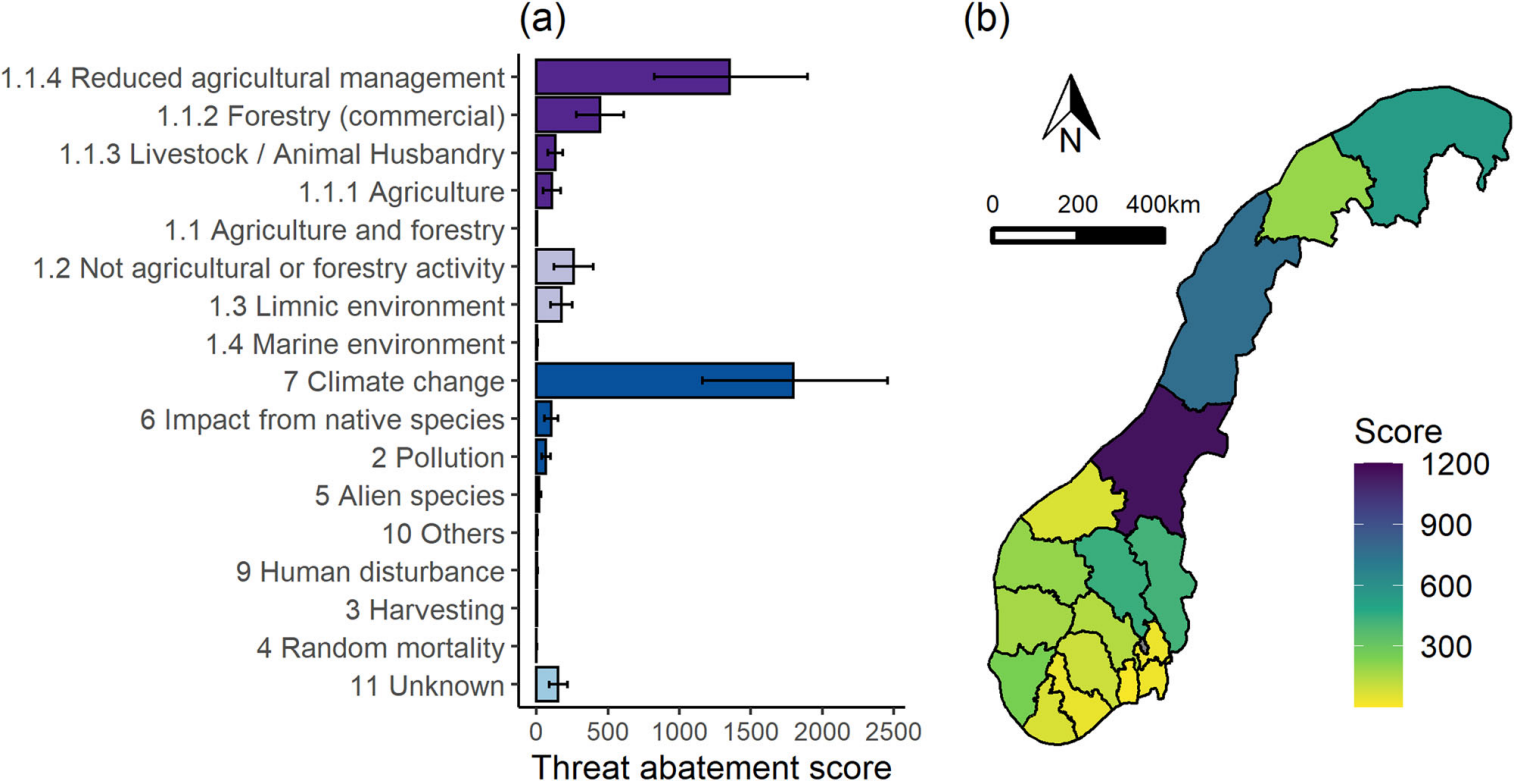
For near‐threatened and threatened species of plants in Norway, (a) threat‐abatement scores for each threat category, which are specific to Norway (Appendix S3) (error bars, maximum and minimum scores due to uncertainty in the proportion of each species’ global distribution in Norway; dark purple, threats to habitat from agriculture and forestry; light purple, threats to habitats from other sources; dark blue, threats that do not affect habitat; light blue, unknown effects of threats), and (b) total threat‐abatement scores per county

The total STAR*_T_* score for plant species in Norway was 4632 (minimum 2810, maximum 6500, which reflected the inclusion of nonendemic species and thus the minimum and maximum estimated share of each species’ global distribution occurring in Norway). The majority of the total STAR*_T_* score was contributed by habitat impacts from agriculture and forestry (classified at the first level in the threat classification hierarchy) (54% total STAR*_T_*), which was primarily driven by reduced agricultural management (i.e., cessation of traditional management practices that maintain the open, seminatural habitat on which many red‐listed vascular plants rely; 29% total STAR*_T_*) (Figure 2a). A large proportion of the total score was also contributed by climate change (39% total STAR*_T_*).

The total STAR*_T_* scores per county are in Figure 2b; the highest scores were in central and northern Norway, where a higher proportion of threatened species with a large proportion of their global distribution in Norway occur.

### South Africa

The total number of threatened endemic plants in South Africa is 2510. Of these, 1894 species had ongoing or future threats documented and had distribution polygons available and were therefore included in the analyses (1009 vulnerable, 678 endangered, and 207 critically endangered species).

The total STAR*_T_* score for endemic threatened plants in South Africa was 479,300. Based on the approach to calculating restorable AOH used here, the total STAR*_R_* score was 29,102 (i.e., habitat restoration could contribute up to 6.1% of the national STAR*_T_* score).

**FIGURE 3:**
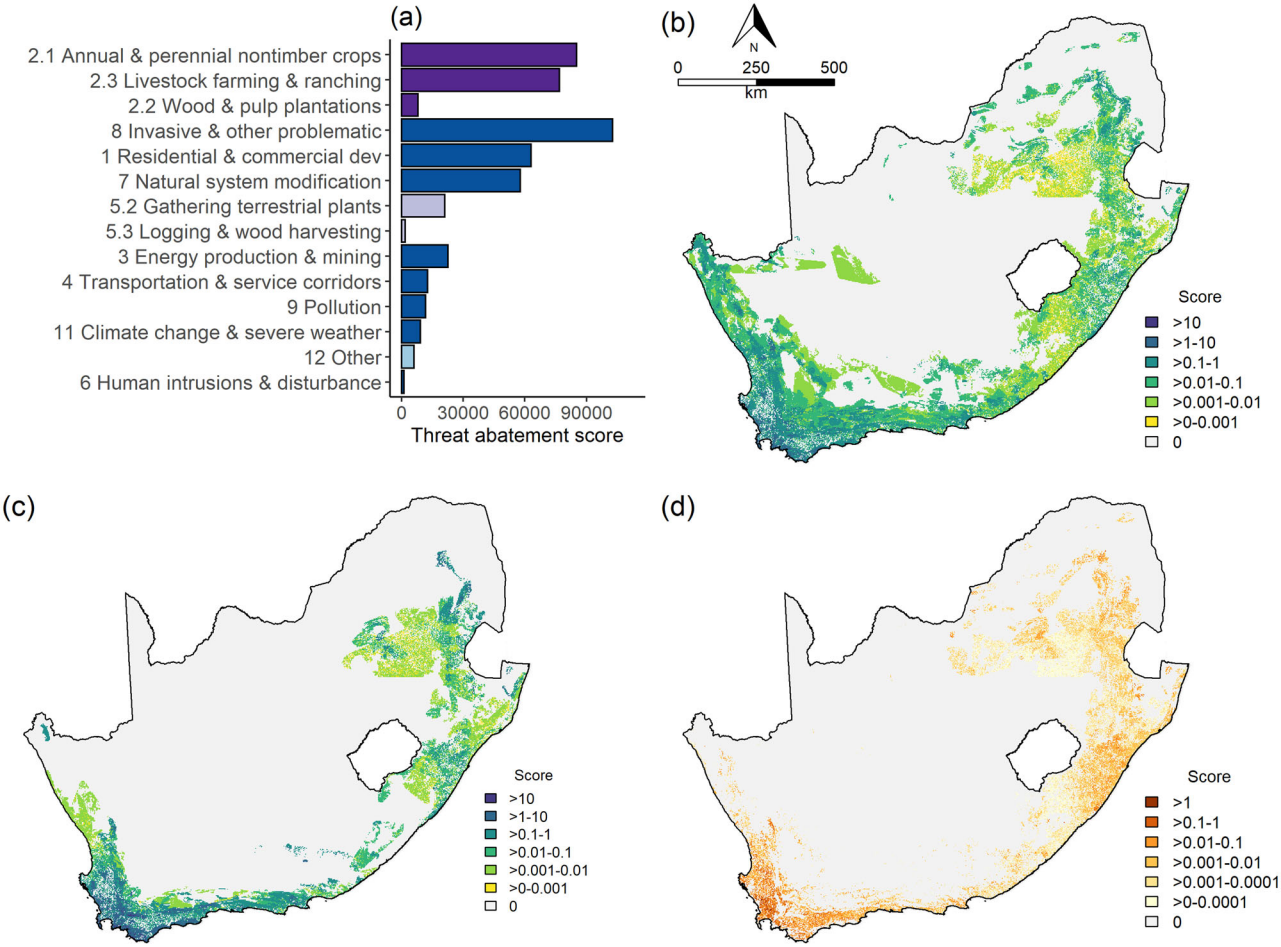
For endemic threatened species of plants in South Africa, (a) threat‐abatement scores per threat category (dark purple, threats from agriculture; light purple, threats from biological resource use; light blue, other threats; dark blue, remaining threats), (b) total threat‐abatement scores, (c) threat‐abatement scores for the threat from invasive non‐native species or disease (demonstrating ability to disaggregate total threat‐abatement score by individual threat), and (d) total restoration scores. Maps (b–d) are 300‐m resolution.

Threats from agriculture contributed 36% to the total threat‐abatement score, with 18% from annual and perennial nontimber crops, 16% from livestock farming and ranching, and 2% from wood and pulp plantations (Figure 3a). This was followed by threats from invasive and other problematic species, which contributed 21% to the total threat‐abatement score, residential and commercial development (13%), and natural system modification (12%).

The mapped total STAR*_T_* scores are in Figure 3b. Overall spatial patterns for threat‐abatement scores closely followed patterns of threatened species richness, such as in the lowlands of the Western Cape and grasslands of the eastern escarpment (Figure 3b). Total threat‐abatement score can be disaggregated by individual threat, and an example for non‐native invasive species is in Figure 3c.

Total STAR*_R_* scores are in Figure 3d. Opportunities for restoration (Figure 3d) indicated that the biggest impact could be achieved in 3 of South Africa's most threatened biomes: Fynbos, grasslands, and the Indian Ocean Coastal Belt. Within these biomes, many threatened species are already below their persistence targets in their remaining intact habitat.

## DISCUSSION

Our 3 case studies showed how the STAR metric can be applied flexibly to national red list data gathered in different ways. Even with different approaches to red list assessments among countries, the STAR metric yielded informative results that quantified the relative contribution of different threats to documented species’ extinction risk and that identified where the greatest conservation gains could be made for species through threat‐abatement activities. Although at a broad scale, regions that emerged with particularly high STAR scores were consistent with those identified in previous analyses (e.g., biodiversity hotspots such as the Atlantic Forest, Cerrado, Cape Floristic Province, Succulent Karoo, and Maputaland‐Pondoland‐Albany [Myers et al., 2000]), STAR enhanced these with explicit quantification and much higher spatial resolution and allowed disaggregation by threats, including spatial mapping of individual threats (as presented for the threat from invasive species in South Africa).

Despite our case study countries being spread across 3 continents, the threat‐abatement scores for red‐listed plant species in Brazil, Norway, and South Africa showed some commonalities. In all cases, the greatest opportunity for reducing plant species’ extinction risk came from abating threats from agricultural activities. Agriculture is the major threat to plants globally (Nic Lughadha et al., 2020) and affects a large proportion of vertebrate species (Maxwell et al., 2016); abating threats from agriculture provides the greatest opportunity to reduce the extinction risk of amphibians, birds, and mammals globally (Mair et al., 2021). The relative contribution of other threats to plant species’ extinction risk varied among countries.

In South Africa, tackling the threat from invasive species provided an opportunity to reduce species’ extinction risk substantially. South Africa has an established program for invasive species control (Wilson et al., 2013), for which STAR analyses could inform target setting. In Norway, the high score for threats from climate change reflected the occurrence of alpine species with relatively large proportions of their global distribution in Norway and poses particular challenges for conservation, given that climate change cannot necessarily be tackled locally (Kyrkjeeide et al., 2021). The results for Brazil highlighted the potential benefit of reducing the impact of urban and agricultural expansion, particularly in the Atlantic Forest and the Cerrado biomes (where forest cover has been reduced to 29% and 54%, respectively, of its original area [Projeto MapBiomas, 2020]), where the majority of assessed species occur. The STAR analysis also identified the increasing threat from changes in fire regimes in Brazil, which are driven by climate change (Jolly et al., 2015) and deforestation (Barlow et al., 2020). The STAR analyses could be used to identify areas where tackling the threat from deforestation could result in the greatest reduction in species’ extinction risk, adding value to the avoidance of deforestation beyond carbon emission considerations.

Our results qualitatively reflect outcomes from other studies, including the finding that agriculture was the greatest threat to biodiversity in Brazil (Bernard et al., 2019). In South Africa, major threats from habitat loss and degradation and from invasive species have been identified elsewhere (Skowno et al., 2019). Similarly, in Norway land‐use change was identified as the major threat (Kyrkjeeide et al., 2021). The STAR metric builds on such previous studies by quantifying the potential relative impact of abating these threats on species’ extinction risk reduction and identifying the places where threat abatement activities could provide the greatest benefits to species.

### Linking national and global policy

Application of STAR has the potential to support direct linkages between national and global policy processes. All 3 countries are Parties to the CBD. The STAR metric could provide a unified conceptual approach to global and national target setting, ensuring that targets and planning for conservation interventions at the national level are aligned with the GBF. Strong and consistent translation from global to national targets will be crucial for successful national‐level implementation of the framework (Xu et al., 2021). By using STAR to identify opportunities to reduce species’ extinction risk nationally, Parties to the CBD could set threat reduction targets to meet GBF action targets and outcome goals. For example, a country could use STAR analyses to inform a target for controlling or eradicating particular invasive species in a particular place, contributing to the proposed GBF invasive species target and the outcome goal for species conservation (CBD, 2021). Similarly, the restoration component of STAR (as applied in the South Africa case study) identifies where habitat restoration activities could best contribute to the reduction of species’ extinction risk and could allow species conservation needs to be incorporated into restoration target setting in response to the UN Decade on Ecosystem Restoration (UN, 2019).

Use of national red list data in STAR analyses offers the benefit of expanded taxonomic coverage, in contrast to the global application of STAR, which is currently limited to terrestrial amphibians, birds, and mammals. Applying STAR to national red list data allows countries to set quantitative conservation targets based on the taxonomic groups of national value or that are particularly well‐known and to harness a broad community to respond to outputs. In countries with broad taxonomic coverage in the national red list, such as Norway, further expansion of STAR could provide the opportunity for often understudied taxonomic groups such as bryophytes and lichens to be considered as well. Furthermore, the much greater number of plant species compared with the number of species in the more commonly studied vertebrate groups produced very large national threat‐abatement scores in our case study countries. In South Africa, the score for plants was 30 times larger than that for vertebrates. The inclusion of a greater diversity of taxonomic groups will provide a deeper understanding of threats to biodiversity. For example, threat‐abatement scores in South Africa demonstrated a greater impact of invasive species on plants than on vertebrates (Mair et al., 2021).

### Challenges and assumptions

There are fundamental assumptions and limitations to our analyses. Due to data limitations, we could not account for spatial variation in species’ population density. Similarly, the metric does not currently reflect spatial variation in threat severity within species’ ranges, a limitation that could be addressed using threat mapping. Missing information on threat scope, severity, and timing resulted in simplified assumptions about the data. We used all available red list assessments, regardless of assessment date; thus, some data may already be out of date. Achieving up‐to‐date and comprehensive assessments of species groups is a major challenge in megadiverse countries. Brazil has assessed >7800 plant species (ca. 31% of its endemic species), equivalent to around 22% of its native terrestrial flora (Brazil Flora Group, 2022). This huge effort provides an as‐yet incomplete understanding of threats to the majority of species. The task of completing assessments is immense. However, the South African experience demonstrates that with appropriate capacity, conservation assessments can be performed relatively affordably (Raimondo et al., 2013).

Variation in red‐listing approaches among countries resulted in variation in data and resulting STAR analyses. We assumed that species range boundaries are precise and known. However, the accuracy of species’ EOO polygons in Brazil is affected by spatial variation in sampling effort; species observations were often recorded along access routes. Meanwhile, the occurrence of species in Norway was recorded to county level, giving coarse spatial data that could not be used to map species AOH. The collection of fine‐scale, accurate species distribution data is a critical action to improve the usefulness and reliability of national STAR analyses, and to enhance the inclusion of species in conservation planning exercises generally. Coarse maps may be useful for framing broad policy responses, but have limited ability to guide site‐level conservation actions. Future studies should explore the optimal spatial resolution of species and threat data for STAR to usefully inform conservation action at site level.

Species distribution data for South Africa were considered reliable; thus, this was the only case study where we were able to apply the restoration component of STAR. We improved the global approach to calculating STAR*_R_* by identifying restorable AOH based on habitat types with reasonable potential for restoration to natural habitat; thus, we avoided the problem of shifting baselines and instead focused on feasibility. The resulting potential contribution of restoration in South Africa was therefore small (only ∼6% of extinction risk reduction can be achieved through habitat restoration), which was also due to the relatively low extent of natural habitat loss in the country (estimated 22% of natural habitat in South Africa has been lost since European arrival [Skowno et al., 2021]). We assumed that all restorable habitat types held equal restoration potential. This simplified assumption could be addressed by developing a restoration multiplier specific to each habitat type, which we suggest is best done at the local scale for site‐specific STAR applications.

### Recommendations and future directions

One of STAR's principal strengths is that the metric, if applied consistently, can be disaggregated across space and taxonomic groups and, conversely, aggregated up from local to national to global scales, allowing the sum of conservation actions across sites to be recognized as a contribution to global species’ extinction risk reduction (Mair et al., 2021) and international commitments under the CBD. This crucial property can be retained in application of STAR to national red list data through restriction to national endemic species (as we did for Brazil and South Africa) (notwithstanding that this will give underestimates of STAR scores for the country as a whole) or through weighting by proportion of global range within the country (as we did for Norway). Where nonendemic species are included in national applications of STAR, the national red list status of species may vary among countries, making comparisons among countries and aggregation to global scales more difficult. However, inclusion of nonendemic species may give a more comprehensive view of national threat‐abatement opportunities, particularly for countries with few endemic species.

The ability to relate national STAR analyses directly to the global context further depends on the rigor and consistency of national red list data and subsequent consistency in metric application. For a STAR analysis to be as informative as possible nationally, and also globally scalable, national red list assessments should be comprehensive for the taxonomic group, follow the updated IUCN guidelines, document all aspects of threats, and include accurate, fine‐scale species’ distribution maps. Strategic reassessments are vital to keep STAR up to date.

We believe the STAR metric fills a niche in species conservation planning and policy. The metric should not be interpreted as providing conservation priorities, for which other methods (e.g., systematic conservation planning) are more appropriate, although such methods could make use of STAR analyses alongside additional data (e.g., socioeconomic). Instead, STAR has been designed to help focus action in a spatially explicit way and to allow diverse actors to set and monitor progress toward species conservation targets. The STAR metric centers on conservation actions, changes in which can be measured over shorter time frames compared with changes in species populations and distributions, such as those measured by the IUCN Red List Index (Stevenson et al., 2021). However, to realize the full potential of STAR, future studies should explore the effort required to realize reductions in species’ extinction risk through threat abatement and restoration activities and the sensitivity of the metric to track changes in response to such actions. We require an understanding of how realistic conservation targets are and which actions can feasibly support target achievement. Finally, STAR is based on the assumption that action can be taken to abate threats at the site level, which is not the case for threats such as climate change. Future development of STAR could consider integrating potential habitat maps to account for climate change impacts on species in a spatially explicit way.

The GBF will set goals and targets at the global level, but action to protect and restore biodiversity will happen at national and local levels. By extending STAR to national red lists in 3 contrasting national contexts, we have shown that STAR provides a mechanism to link national implementation to global aspiration. Quantifiably linking local taxonomic coverage and data collection to global processes in this way will allow national target setting to align with global targets and enable nonstate actors to measure and report on their own potential contributions to species conservation. Engaging multiple sectors and levels of government in a unified and quantitative way is critical for enabling and measuring the transformative change required to realize the ambitious Convention on Biological Diversity's 2050 Vision of Living in Harmony with Nature.

## Supporting information

Supporting Information
Additional supporting information may be found in the online version of the article at the publisher's website.
