# Supplementary material for: Quantifying and mapping species threat abatement opportunities to support national target setting
Source: Conserv Biol. 2022 Dec 13;37(1):e14046. doi: 10.1111/cobi.14046 (PMC10108230; doi:10.1111/cobi.14046)
Supplement: Supplementary file 1 — Supporting Information Additional supporting information may be found in the online version of the article at the publisher's website. [file COBI-37-0-s001.docx]

# Quantifying and mapping species threat abatement opportunities to support national target-setting

# Supporting Information

**Appendix S1.** Example calculation of the Species Threat Abatement and Restoration (STAR) metric.

**Appendix S2.** Crosswalk of the Brazil threat classification system to the IUCN threat classification system.

**Appendix S3.** Translation of the Norway national red list threat categories from Norwegian to English.

**Appendix S4**. The percentage bands used to estimate the share of each species’ global distribution occurring in Norway.

**Appendix S5.** Classification of land cover change categories in South Africa.

**Appendix S6**. South Africa current natural habitat and restorable habitat maps.

## Appendix S1

### Example calculation of the Species Threat Abatement and Restoration (STAR) metric

As stated in the main text, the STAR threat-abatement score (*T*) for a location (*i*) and threat (*t*) is calculated among all species as (formula 1):

$$T_{t,i}= \sum_{s}^{N_{s}} P_{s,i}W_{s}C_{s,t}$$

where *P*_s,i_ is the current Area of Habitat (AOH; Brooks et al. 2019) of each species (*s*) within location (*i*), expressed as a percentage of the global species’ current AOH; W_s_ is the IUCN Red List category weight of species *s* (Near Threatened = 1, Vulnerable = 2, Endangered = 3 and Critically Endangered = 4); C is the relative contribution of threat *t* to the extinction risk of species *s*; and N_s_ is the total number of species at location (*i*). The relative contribution of each threat (following a standard threats classification scheme; Salafsky et al. 2008) to the species’ extinction risk was calculated as the percentage population decline from that threat (derived from the product of severity and scope for that threat in each species’ IUCN Red List assessment) divided by the sum of percentage population declines from all threats to that species.

Taking a hypothetical example, we have a country with three endemic species (A, B and C; Table S1). Being endemic, each species therefore has 100% of their current Area of Habitat (AOH) within the country. The species vary in extinction risk, and so their respective species weighting varies accordingly (Table S1). There are only two threats operating (threat 1 and threat 2), and the scope and severity of these threats vary among species (Table S1). Threat scope and severity are used to calculate the expected population decline caused by that threat; details of how these data are combined are presented in Mair et al. (2021), but in principle the severity of the population decline caused by the threat (e.g. “Rapid declines”, as for Species C, are 20-30% over ten years or three generations, whichever is longest) is weighted by the scope of the threat (e.g. “Majority”, as for Species C, is 50-90% of the species population) to give the overall expected impact of the threat on the entire species population.

We use the expected population decline caused by each threat to calculate the relative contribution of each threat to species extinction risk (*C_s,t_*), by dividing the percentage population decline caused by each threat, by the sum of percentage population declines from all threats to that species. For example, for Species B, the relative contribution of threat 1 is: 10 / (10+5) = 66.6% (Table S2).

We can then calculate the score per threat per species as: the percent of species current AOH present (*P_s,i_*) multiplied by the species weight (*W_s_*) multiplied by the relative contribution of the threat (*C_s,t_*). So for Species B, the score for threat 1 is: 100 x 3 x 66.6 = 200 (Table S2).

The total score for each species is obtained by summing scores across all threats for that species (in this case Species A, B and C score 200, 300 and 400 respectively; directly related to their extinction risk). The total score for each threat is achieved by summing scores across all species for that threat (threats 1 and 2 score 600 and 300 respectively; Table S2). The total score for the country is obtained by summing total scores across all species present (country total is 900; Table S2).

**Table S1**. Data for a hypothetical country example with three endemic species (A, B and C), each with varying extinction risk. Two threats operate (threats 1 and 2) and the scope and severity of these threats vary among species. The expected percentage population decline caused by each threat is calculated based on the combination of scope and severity score (for details see Mair et al. 2021). The same notation as used in formula 1 is presented here to indicate which data are required for each component of the formula.

|  | Formula component | | | | | | | | |
| --- | --- | --- | --- | --- | --- | --- | --- | --- | --- |
|  | *P*_s,I_ | W_s_ | | C_s,t_ | | | | | |
|  |  |  | | Threat 1 | | | Threat 2 | | |
| Species | % species current AOH | Species red list category | Species weighting | Scope | Severity | % population decline | Scope | Severity | % population decline |
| A | 100 | VU | 2 | Majority | Slow, significant declines | 9% | - | - | - |
| B | 100 | EN | 3 | Whole | Slow, significant declines | 10% | Minority | Slow, significant declines | 5% |
| C | 100 | CR | 4 | Majority | Rapid declines | 18% | Majority | Rapid declines | 18% |

**Table S2**. STAR threat-abatement calculations for our hypothetical country example, presenting the scores per threat per species, the total scores for each species A, B and C, the summed scores for each threat 1 and 2 in the country, and the total threat-abatement score for the country.

|  | *P*_s,I_ | W_s_ | C_s,t_ | | Scores | | |
| --- | --- | --- | --- | --- | --- | --- | --- |
| Species | % species current AOH | Species weighting | Relative contribution of threat 1 | Relative contribution of threat 2 | Threat 1 score | Threat 2 score | **Species total score** |
| A | 100 | 2 | 100% | 0 | 200 | 0 | **200** |
| B | 100 | 3 | 66.6% | 33.3% | 200 | 100 | **300** |
| C | 100 | 4 | 50% | 50% | 200 | 200 | **400** |
|  |  |  |  | **Threat total** | **600** | **300** | **900** |

### Target setting and progress tracking

Once the STAR score for a particular area has been calculated, threat-abatement targets can be set and progress towards these targets monitored. For example, if the management decision was to eliminate threat 2 from our hypothetical country in Table S2, then the quantitative target would be to realize a STAR_T_ score of 300 (the total STAR_T_ score for threat 2) out of a potential total STAR_T_ country score of 900, i.e. to realize ~33% of the total potential STAR_T_ score. Progress towards this target could be monitored by measuring changes in the intensity of threat 2, until the threat was eliminated.

Upon achievement of this target for threat 2, there would remain conservation opportunities through the abatement of threat 1. It may be, for example, that threat 1 was particularly pervasive and, realistically, could only be reduced rather than eliminated. The target for this threat may therefore be to reduce the threat intensity by a third, i.e. to realize a STAR_T_ score of 200 for threat 2, which would be a further ~22% of the total potential STAR_T_ score for the country. Meeting targets for both threats would therefore result, overall, in realizing 55% of the total potential STAR_T_ for the country.

Progress towards such threat-abatement targets can be measured over relatively short time periods, as action may be planned – and threat intensity change – over a period of years. The expectation is that, with sufficient threat-abatement action across species’ distributions, over the longer term species populations and/or distributions would recover to such an extent that their extinction risk would be reduced and they would be downlisted on the IUCN Red List.

## Appendix S2

Crosswalk of the Brazil threat classification system to the IUCN threat classification system. Only those threat categories relevant to Brazil red list assessment of plants are presented.

| **Brazil threat classification scheme** | | **IUCN threat classification threat code and name** |
| --- | --- | --- |
| **Code** | **Threat name** |  |
| **1** | **Habitat Loss/Degradation (human induced)** | 7.3 Other ecosystem modifications |
| 1.1 | Agriculture | 2 Agriculture & aquaculture |
| 1.1.1 | Crop | 2.1 Annual & perennial non-timber crops |
| 1.1.1.1 | Shifting Agriculture | 2.1.1 Shifting agriculture |
| 1.1.1.2 | Small-holder farming | 2.1.2 Small-holder farming |
| 1.1.1.3 | Agro-industry farming | 2.1.3 Agro-industry farming |
| 1.1.2 | Wood plantation | 2.2 Wood & pulp plantations |
| 1.1.2.1 | Small-scale | 2.2.1 Small-holder plantations |
| 1.1.2.2 | Large-scale | 2.2.2 Agro-industry plantations |
| 1.1.3 | Non-timber plantation | 2.1 Annual & perennial non-timber crops |
| 1.1.3.1 | Small-scale | 2.1.2 Small-holder farming |
| 1.1.3.2 | Large-scale | 2.1.3 Agro-industry farming |
| 1.1.4 | Livestock | 2.3 Livestock farming & ranching |
| 1.1.4.1 | Nomadic | 2.3.1 Nomadic grazing |
| 1.1.4.2 | Small-holder | 2.3.2 Small-holder grazing, ranching or farming |
| 1.1.4.3 | Agro-industry | 2.3.3 Agro-industry grazing, ranching or farming |
| 1.1.5 | Abandonment | 12.1 Other threat |
| 1.1.6 | Marine aquaculture | 2.4 Marine & freshwater aquaculture |
| 1.2 | Land management of non-agricultural area | 12.1 Other threat |
| 1.2.1 | Abandonment | 12.1 Other threat |
| 1.2.2 | Change of management regime | 12.1 Other threat |
| 1.3 | Extraction | 7.3 Other ecosystem modifications |
| 1.3.1 | Mining | 3.2 Mining & quarrying |
| 1.3.2 | Fishing | 5.4 Fishing & harvesting aquatic resources |
| 1.3.3 | Wood | 5.3 Logging & wood harvesting |
| 1.3.3.1 | Small-scale subsistence | 5.3.5 Motivation Unknown/Unrecorded |
| 1.3.3.2 | Selective logging | 5.3.5 Motivation Unknown/Unrecorded |
| 1.3.3.3 | Clear-cutting | 5.3.5 Motivation Unknown/Unrecorded |
| 1.3.4 | Non-woody vegetation collection | 5.2.4 Motivation Unknown/Unrecorded |
| 1.3.6 | Groundwater extraction | 7.2.8 Abstraction of ground water (unknown use) |
| 1.3.8 | Unknown | 7.3 Other ecosystem modifications |
| 1.4 | Infrastructure development | 1 Residential & commercial development |
| 1.4.1 | Industry | 1.2 Commercial & industrial areas |
| 1.4.2 | Human settlement | 1.1 Housing & urban areas |
| 1.4.3 | Tourism/recreation | 1.3 Tourism & recreation areas |
| 1.4.4 | Transport - land/air | 4.1 Roads & railroads |
| 1.4.5 | Transport - water | 4.3 Shipping lanes |
| 1.4.6 | Dam | 7.2.11 Dams (size unknown) |
| 1.4.7 | Telecommunication | 4.2 Utility & service lines |
| 1.4.8 | Power line | 4.2 Utility & service lines |
| 1.5 | Invasive alien species (directly impacting habitat) | 8.4 Problematic species/diseases of unknown origin |
| 1.6 | Change in native species dynamics (directly impacting habitat | 6.3 Work & other activities |
| 1.7 | Fire | 7.1 Fire & fire suppression |
| **2** | **Invasive alien species (directly affecting the species)** | 8 Invasive & other problematic species, genes & diseases |
| 2.1 | Competitor | 8.1 Invasive non-native/alien species/diseases |
| 2.2 | Predator | 8.1 Invasive non-native/alien species/diseases |
| 2.3 | Hybridizer | 8.1 Invasive non-native/alien species/diseases |
| 2.4 | Pathogens/parasite | 8.4 Problematic species/diseases of unknown origin |
| **3** | **Harvesting (hunting/gathering)** | 5.2 Gathering terrestrial plants |
| 3.1 | Foods | 5.2 Gathering terrestrial plants |
| 3.1.1 | Subsistence use/local trade | 5.2 Gathering terrestrial plants |
| 3.1.2 | Sub-national/national trade | 5.2 Gathering terrestrial plants |
| 3.1.3 | Regional/international trade | 5.2 Gathering terrestrial plants |
| 3.2 | Medicinal products | 5.2 Gathering terrestrial plants |
| 3.2.1 | Subsistence use/local trade | 5.2 Gathering terrestrial plants |
| 3.2.2 | Sub-national/national trade | 5.2 Gathering terrestrial plants |
| 3.2.3 | Regional/international trade | 5.2 Gathering terrestrial plants |
| 3.3 | Fuel | 3.1 Oil & gas drilling |
| 3.3.1 | Subsistence use/local trade | 3.1 Oil & gas drilling |
| 3.3.2 | Sub-national/national trade | 3.1 Oil & gas drilling |
| 3.4 | Material | 5.3 Logging & wood harvesting |
| 3.4.1 | Subsistence use/local trade | 5.3 Logging & wood harvesting |
| 3.4.2 | Sub-national/national trade | 5.3 Logging & wood harvesting |
| 3.4.3 | Regional/international trade | 5.3 Logging & wood harvesting |
| 3.5 | Cultural/scientific/leisure activities | 5.2 Gathering terrestrial plants |
| 3.5.1 | Subsistence use/local trade | 5.2 Gathering terrestrial plants |
| 3.5.2 | Sub-national/national trade | 5.2 Gathering terrestrial plants |
| 3.5.3 | Regional/international trade | 5.2 Gathering terrestrial plants |
| **6** | **Pollution (affecting habitat and/or species)** | 9 Pollution |
| 6.1 | Atmospheric pollution | 9.5 Air-borne pollutants |
| 6.1.1 | Global warming/oceanic warming | 11.5 Other impacts |
| 6.2 | Land pollution | 9 Pollution |
| 6.2.1 | Agriculture | 9.3 Agricultural & forestry effluents |
| 6.3 | Water pollution | 9 Pollution |
| 6.3.1 | Agriculture | 9.3 Agricultural & forestry effluents |
| 6.3.2 | Domestic | 9.1 Domestic & urban waste water |
| 6.3.3 | Commercial/Industrial | 9.2 Industrial & military effluents |
| **7** | **Natural disaster** | 7.3 Other ecosystem modifications |
| 7.2 | Storms/flooding | 11.4 Storms & flooding |
| 7.3 | Temperature extreme | 11.3 Temperature extremes |
| 7.4 | Wildfire | 7.1 Fire & fire suppression |
| 7.6 | Avalanches/landslide | 10.3 Avalanches/landslides |
| 7.7 | Other | 12.1 Other threat |
| **8** | **Changes in native species dynamic** | 12.1 Other threat |
| 8.1 | Competitor | 12.1 Other threat |
| 8.2 | Predator | 12.1 Other threat |
| 8.3 | Prey/food base | 12.1 Other threat |
| 8.5 | Pathogens/parasite | 12.1 Other threat |
| 8.6 | Mutualism | 12.1 Other threat |
| 8.7 | Other | 12.1 Other threat |
| **9** | **Intrinsic factor** | 12.1 Other threat |
| 9.1 | Limited dispersal | 12.1 Other threat |
| 9.2 | Poor recruitment/reproduction/regeneration | 12.1 Other threat |
| 9.3 | High juvenile mortality | 12.1 Other threat |
| 9.4 | Inbreeding | 12.1 Other threat |
| 9.5 | Low densitie | 12.1 Other threat |
| 9.8 | Population fluctuation | 12.1 Other threat |
| 9.9 | Restricted range | 12.1 Other threat |
| 9.10 | Other | 12.1 Other threat |
| **10** | **Human disturbance** | 6 Human intrusions & disturbance |
| 10.1 | Recreation/tourism | 6.1 Recreational activities |
| 10.3 | War/civil unrest | 6.2 War, civil unrest & military exercises |
| 10.4 | Transport | 4.1 Roads & railroads |
| 10.5 | Fire | 7.1 Fire & fire suppression |
| 10.6 | Other | 12.1 Other threat |
| **11** | **Other** | 12.1 Other threat |
| **12** | **Unknown** | 12.1 Other threat |

## Appendix S3

Translation of the Norway national red list threat categories from Norwegian to English, and the numeric codes assigned to each category to denote the classification hierarchy.

| **Pavirkningsfaktor (Norwegian threat name)** | **Code** | **English Threat Name** |
| --- | --- | --- |
| Ingen trussel | 0 | No threat |
| **Påvirkning på habitat** | **1** | **Impact on habitat** |
| Landbruk | 1.1 | Agriculture and forestry |
| Jordbruk | 1.1.1 | Agriculture |
| Oppdyrking | 1.1.1.1 | Cultivation |
| Drenering (grøfting) | 1.1.1.2 | Drainage (ditching) |
| Irrigasjon | 1.1.1.3 | Irrigation |
| Torvbryting | 1.1.1.4 | Peat mining |
| Slått | 1.1.1.5 | Mowing |
| Endrede frøblandinger | 1.1.1.6 | Changed seed mixtures |
| Motorferdsel | 1.1.1.7 | Motor traffic |
| Andre | 1.1.1.8 | Others |
| Skogbruk (kommersielt) | 1.1.2 | Forestry (commercial) |
| Skogsdrift, hogst og skjøtsel | 1.1.2.1 | Forestry, harvesting and management |
| Åpne hogstformer (flatehogst og frøtrehogst som også inkluderer uttak av rotvelt, råtne trær, tørrgran etc.) | 1.1.2.1.1 | Open harvesting forms (clear-cutting, cutting of crop trees, which also includes windfalls, rotten trees, dry spruce, etc.) |
| Lukkede hogstformer (plukkhogst, skjermstilling, tynning, uttak av enkelttrær, inkludert uttak av rotvelt, råtne trær, tørrgran etc.) | 1.1.2.1.2 | Closed harvesting forms (selection felling, screen positioning, thinning, felling of single trees, including windfalls, rotten trees, dry spruce, etc.) |
| Ungskogrydding (rydding i ungskog) | 1.1.2.1.3 | Young forest clearing (clearing in young forest) |
| Uttak av død ved (stående *gadd* og liggende *læger*) | 1.1.2.1.4 | Withdrawal of dead wood (standing and lyingdead wood |
| Markberedning (maskinell bearbeiding av marksjiktet etter hogst) | 1.1.2.1.5 | Soil preparation (machining of the soil layer after harvesting) |
| Gjødsling | 1.1.2.1.6 | Fertilization |
| Sprøyting (av plantefelt for fjerning av løv og gras) | 1.1.2.1.7 | Spraying (of plant fields for the removal of leaves and grass) |
| Skogsbilveger og kjørespor etter skogsmaskiner (den direkte effekten av inngrepet) | 1.1.2.1.8 | Forest roads and tyre marks after forest machines (the direct effect of the intervention) |
| Grøfting og grøfterens (f.eks. myr og sumpskog) | 1.1.2.1.9 | Ditching and cleaning of ditches (eg bog and swamp forest) |
| Skogreising (aktiv gjenplanting av tidligere åpen mark) | 1.1.2.1.10 | Afforestation (active replanting of previously open land) |
| Treslagsskifte (gran på Vestlandet og nord for Saltfjellet, fremmede treslag) | 1.1.2.1.11 | Tree species transformation (spruce in western Norway and north of Saltfjellet, foreign species) |
| Skogsdrift, hogst og skjøtsel > Skogbrannslukking | 1.1.2.1.12 | Forest fire fighting |
| Andre faktorer | 1.1.2.1.13 | Other factors |
| Skogreising/treslagskifte | 1.1.2.2 | Afforestation / tree species transformation |
| Treslagsskifte (gran på Vestlandet og nord for Saltfjellet, fremmede treslag) | 1.1.2.2.1 | Tree species transformation (spruce in western Norway and north of Saltfjellet, foreign wood species) |
| Skogreising (aktiv gjenplanting av tidligere åpen mark) | 1.1.2.2.2 | Afforestation (active replanting of previously open land) |
| Grøfting og grøfterens (f.eks. myr og sumpskog) | 1.1.2.2.3 | Ditching and cleaning of ditches (eg bog and swamp forest) |
| Andre faktorer | 1.1.2.2.4 | Other factors |
| Skogbrannslukking | 1.1.2.3 | Forest fire fighting |
| Buskap/dyrehold | 1.1.3 | Livestock / Animal Husbandry |
| Tråkk | 1.1.3.1 | Trampling |
| Beite | 1.1.3.2 | Grazing |
| Andre | 1.1.3.3 | Others |
| Opphørt/redusert drift | 1.1.4 | Reduced agricultural management |
| Slått | 1.1.4.1 | Mowing |
| Beite | 1.1.4.2 | Grazing |
| Tråkk og motorferdsel | 1.1.4.3 | Trampling and motor traffic |
| Lyngbrenning | 1.1.4.4 | Heather burning |
| Plukkhogst | 1.1.4.5 | Harvesting/selection felling |
| Tynning | 1.1.4.6 | Thinning |
| Vedhogst | 1.1.4.7 | Logging |
| Styving | 1.1.4.8 | Pollarding |
| Endret bygningstruktur (mangel på høyløer, staller, jordkjellere, rest mm.) | 1.1.4.9 | Changed building structure (lack of hay feeds, stables, cellars, residue etc.) |
| Andre | 1.1.4.10 | Others |
| Habitatpåvirkning - ikke jord- eller skogbruksaktivitet (terrestrisk) | 1.2 | Habitat impact - not agricultural or forestry activity (terrestrial) |
| Utbygging/utvinning | 1.2.1 | Development / extraction |
| Infrastruktur (veier, broer, flyplasser mm.) | 1.2.1.1 | Infrastructure (roads, bridges, airports etc.) |
| Industri/næringsutbygging | 1.2.1.2 | Industry / industrial development |
| Boligbebyggelse/boligutbygging | 1.2.1.3 | Residential development / housing development |
| Turisme/rekreasjon (parker, idrettsanlegg, stier/løyper mm.) | 1.2.1.4 | Tourism / recreation (parks, sports facilities, trails, etc.) |
| Utvinning (gruvedrift og steinbrudd) | 1.2.1.5 | Extraction (mining and quarrying) |
| Masseuttak (leire, sand og grustak) | 1.2.1.6 | Bulk extraction (clay, sand and gravel pits) |
| Deponering (dumping, utfyllinger og avfallsdeponier) | 1.2.1.7 | Landfill (dumping, landfills and waste disposal sites) |
| Kraftledninger | 1.2.1.8 | Power lines |
| Vindkraftutbygging | 1.2.1.9 | Wind power development |
| Rassikring | 1.2.1.10 | Landslide protection |
| Andre | 1.2.1.11 | Other |
| Opphørt drift | 1.2.2 | Discontinued operation |
| Opphør av masseuttak (leire, sand og grustak) | 1.2.2.1 | Cessation of bulk extraction (clay, sand and gravel pits) |
| Opphørt tråkk og motorferdsel | 1.2.2.2 | Discontinued trampling and motor traffic |
| Endring i avfallshåndtering (mangel på kompost, sagflis, slaktavfall mm.) | 1.2.2.3 | Change in waste management (lack of compost, sawdust, offal etc.) |
| Endret bygningstruktur | 1.2.2.4 | Changed building structure |
| Andre | 1.2.2.5 | Other |
| Annen påvirkning på habitat | 1.2.3 | Other impact on habitat |
| Uttak av død ved (stående *gadd* og liggende *læger*) | 1.2.3.1 | Withdrawal of dead wood (standing and lying dead wood ) |
| Vedhogst, avvirkning av spesielle type trær (gamle, hule, brannskade) | 1.2.3.2 | Logging, felling of special trees (old, hollow, burned) |
| Forbedret hygiene innendørs | 1.2.3.3 | Improved indoor hygiene |
| Branner | 1.2.3.4 | Fires |
| Motorferdsel | 1.2.3.5 | Motor traffic |
| Andre | 1.2.3.6 | Other |
| Habitatpåvirkning i limnisk miljø | 1.3 | Habitat impact in limnic environment |
| Ferskvannsakvakultur | 1.3.1 | Freshwater aquaculture |
| Mudring, dumping og utfyllinger i strandsonen | 1.3.2 | Sewage, dumping and beach zone fillings |
| Oppdemming/vannstandsregulering/overføring av vassdrag | 1.3.3 | Drainage / water level regulation / transfer of watercourses |
| Vannløpsendring (flomhindring, kanalisering, utretting, moloer, terskler mm.) | 1.3.4 | Water flow change (flood prevention, channelization, straightening, molos, thresholds, etc.) |
| Gjennfylling av dammer, bekkelukking og tørrlegging | 1.3.5 | Pond filling, brook closure and drying |
| Andre | 1.3.6 | Others |
| Habitatpåvirkning i marine miljø | 1.4 | Habitat impact in marine environment |
| Marin akvakultur | 1.4.1 | Marine aquaculture |
| Mudring, dumping og utfyllinger i strandsonen (inkl. moloer og havneanlegg) | 1.4.2 | Dredging, dumping and landfills in the tidal zone (incl. Piers and port facilities) |
| Åpning av innløp til poller | 1.4.3 | Opening of inlets to fjords |
| Bunntråling | 1.4.4 | Bottom trawling |
| Taretråling/tangskjering | 1.4.5 | Tare trawling / seaweed cutting |
| Petroleumsaktivitet (oljeboring, rørlegging, oppankring mm.) | 1.4.6 | Petroleum activity (oil drilling, piping, anchoring, etc.) |
| Andre | 1.4.7 | Others |
| **Forurensing** | **2** | **Pollution** |
| Terrestrisk | 2.1 | Terrestrial |
| Næringssalter og organiske næringsstoffer | 2.1.1 | Nutrients and organic nutrients |
| Biocider | 2.1.2 | Biocides |
| Uorganiske gifter (tungmetaller, arsen, fluor mm.) | 2.1.3 | Inorganic toxins (heavy metals, arsenic, fluorine, etc.) |
| Organiske gifter (PAH mm.) | 2.1.4 | Organic toxins (PAH etc.) |
| Radioaktivitet | 2.1.5 | Radioactivity |
| Tilsetninger i dyrefor/medisinering | 2.1.6 | Additives in animal feed / medication |
| Andre | 2.1.7 | Others |
| I vann | 2.2 | In water |
| Næringssalter og organiske næringstoffer | 2.2.1 | Inorganic and organic nutrients |
| Biocider | 2.2.2 | Biocides |
| Uorganiske gifter (tungmetaller, arsen, fluor mm.) | 2.2.3 | Inorganic toxins (heavy metals, arsenic, fluorine etc.) |
| Organiske gifter (PAH mm.) | 2.2.4 | Organic toxins (PAH etc.) |
| Radioaktivitet | 2.2.5 | Radioactivity |
| Oljeutslipp | 2.2.6 | Oil spills |
| Sur nedbør | 2.2.7 | Acid rainfall |
| Kalking | 2.2.8 | Liming |
| Tilsetninger i dyrefor/medisinering | 2.2.9 | Additives in animal feed / medication |
| Andre | 2.2.10 | Other |
| Atmosfærisk | 2.3 | Atmospheric |
| Bakkenært ozon | 2.3.1 | Ground-level ozone |
| NOx-forbindelser | 2.3.2 | NOx compounds |
| Forsurende gasser (S-forbindelser) | 2.3.3 | Acidifying gases (S-compounds) |
| Ozonnedbrytende gasser (indirekte effekter av UV stråling) | 2.3.4 | Ozone-depleting gases (indirect effects of UV radiation) |
| Utslipp av klimagasser (CO2), indirekte effekter | 2.3.5 | Greenhouse gas emissions (CO2), indirect effects |
| Andre | 2.3.6 | Other |
| **Høsting** | **3** | **Harvesting** |
| Regulert jakt, fangst eller fiske | 3.1 | Regulated hunting, trapping or fishing |
| Uregulert jakt, fangst eller fiske | 3.2 | Unregulated hunting, trapping or fishing |
| Flora-/faunakriminalitet | 3.3 | Flora / fauna crime |
| Sanking/høsting | 3.4 | Gathering / harvesting |
| Indirekte via høsting av artens næring | 3.5 | Indirectly via harvesting of the species' food/nourishment |
| Andre | 3.6 | Others |
| **Tilfeldig mortalitet** | **4** | **Random mortality** |
| Kollisjoner | 4.1 | Collisions |
| Ledninger, master, bygninger | 4.1.1 | Wires, masts, buildings |
| Vindmøller | 4.1.2 | Wind turbines |
| Kjøretøy | 4.1.3 | Vehicles |
| Andre | 4.1.4 | Others |
| Bifangst | 4.2 | By-catch |
| Fiskerelatert | 4.2.1 | Fishing related |
| Kroking | 4.2.1.1 | Hooking |
| Garnfangst | 4.2.1.2 | Yarn catch |
| Tråling | 4.2.1.3 | Trawling |
| Dynamitt | 4.2.1.4 | Dynamite |
| Forgiftning | 4.2.1.5 | Poisoning |
| Terrestrisk | 4.2.2 | Terrestrial |
| Feller, snarer, nett | 4.2.2.1 | Traps, snares, nets |
| Skyting | 4.2.2.2 | Shooting |
| Forgiftning | 4.2.2.3 | Poisoning |
| Andre | 4.3 | Others |
| Andre | 4.3.1 | Others |
| **Fremmede arter** | **5** | **Alien species** |
| Konkurrenter | 5.1 | Competitors |
| Predatorer | 5.2 | Predators |
| Hybridisering | 5.3 | Hybridization |
| Patogener/parasitter | 5.4 | Pathogens / parasites |
| Påvirker habitatet | 5.5 | Affects the habitat |
| Andre | 5.6 | Others |
| **Påvirkning fra stedegne arter** | **6** | **Impact from native species** |
| Konkurrenter | 6.1 | Competitors |
| Predatorer | 6.2 | Predators |
| Byttedyr/næringskilde | 6.3 | Prey / food source |
| Hybridisering | 6.4 | Hybridization |
| Patogener/parasitter | 6.5 | Pathogens / parasites |
| Mutualisme | 6.6 | Mutualism |
| Påvirker habitatet (beite tråkk mm.) | 6.7 | Affects the habitat (grazing, trampling etc.) |
| Andre | 6.8 | Others |
| **Klimatiske endringer** | **7** | **Climate change** |
| Regionale | 7.1 | Regional |
| Endringer i nedbørsmengde | 7.1.1 | Changes in rainfall |
| Temperaturendring | 7.1.2 | Temperature change |
| Lokale | 7.2 | Local |
| Endringer i lokale lysforhold | 7.2.1 | Changes in local light conditions |
| Endringer i lokale temperaturforhold | 7.2.2 | Changes in local temperature conditions |
| Endrede lokale fuktighetsforhold | 7.2.3 | Changed local humidity conditions |
| Andre | 7.3 | Others |
| **Naturkatastrofer** | **8** | **Natural Disasters** |
| Tørke | 8.1 | Drought |
| Stormer | 8.2 | Storms |
| Oversvømmelser | 8.3 | Floods |
| Ekstreme temperaturer | 8.4 | Extreme temperatures |
| Branner | 8.5 | Fires |
| Vulkaner | 8.6 | Volcanoes |
| Ras/skred | 8.7 | Avalanche / Landslide |
| Andre | 8.8 | Others |
| **Menneskelig forstyrrelse** | **9** | **Human disturbance** |
| Rekreasjon/turisme | 9.1 | Recreation / Tourism |
| Forskning | 9.2 | Research |
| Krig/sivil uro | 9.3 | War / civil unrest |
| Transport | 9.4 | Transportation |
| Branner | 9.5 | Fires |
| Innsamling | 9.6 | Collection |
| Støy og ferdsel (forstyrrelser i hekketid mm.) | 9.7 | Noise and traffic (disturbance during nesting time, etc.) |
| Andre | 9.8 | Others |
| **Andre** | **10** | **Others** |
| **Ukjent** | **11** | **Unknown** |
| **Påvirkning utenfor Norge** | **12** | **Impact outside Norway** |

## Appendix S4

The percentage bands used to estimate the share of each species’ global distribution occurring in Norway. The minimum, median and maximum share of the species global population present were used to calculate minimum, median and maximum threat abatement scores.

| Share of global/European population category | Minimum share | Median share | Maximum share |
| --- | --- | --- | --- |
| <1% | 0.1% | 0.5% | 0.9% |
| 1-5% | 1% | 3% | 5% |
| 5-25% | 5% | 15% | 25% |
| 25-50% | 25% | 37.5% | 50% |
| >50% | 51% | 75% | 100% |

## Appendix S5

Classification of land cover change categories in South Africa (from a map of land cover change 1990-2014, provided by the South African National Biodiversity Institute and following methods in Skowno et al. 2021) as current natural habitat, restorable habitat, or not restorable habitat. Value is the land cover cell value used to identify of each cell type in the land cover raster, cell count the number of cells per category, and change in land cover are described at the second and third level of detail in the land cover classification hierarchy.

| **Value** | **Cell count** | **Change described at level 2 in land cover classification hierarchy** | **Change described at level 3 in land cover classification hierarchy** | **Classification** |
| --- | --- | --- | --- | --- |
| 3 | 105882535 | Natural - no change | Bare ground to Bare ground | Current natural |
| 37 | 31750559 | Natural - no change | Natural to Bare ground | Current natural |
| 7 | 44346802 | Natural - no change | Bare ground to Natural | Current natural |
| 43 | 890249680 | Natural - no change | Natural to Natural | Current natural |
| 67 | 15327384 | Natural marine - no change | Natural marine to Natural marine | Current natural |
| 64 | 1218558 | Natural shore - no change | Natural shore to Natural shore | Current natural |
| 39 | 2214797 | Lost post 1990 | Natural to Cropland - irrigated | Restorable |
| 40 | 16257185 | Lost post 1990 | Natural to Cropland - not irrigated | Restorable |
| 5 | 7847 | Lost post 1990 | Bare ground to Erosion | Restorable |
| 41 | 756330 | Lost post 1990 | Natural to Erosion | Restorable |
| 6 | 9073 | Lost post 1990 | Bare ground to Mine | Restorable |
| 33 | 2119391 | Lost pre 1990 - no change | Mine to Mine | Restorable |
| 42 | 813881 | Lost post 1990 | Natural to Mine | Restorable |
| 63 | 388 | Lost shore post 1990 | Natural shore to Mine shore | Restorable |
| 8 | 3008 | Lost pre 1990 - no change | Bare ground to Plantation | Restorable |
| 34 | 9320 | Lost pre 1990 - no change | Mine to Plantation | Restorable |
| 44 | 3317110 | Lost post 1990 | Natural to Plantation | Restorable |
| 15 | 2543369 | Lost pre 1990 - some recovery | Built up to Secondary natural - post 1990 | Restorable |
| 23 | 14940591 | Lost pre 1990 - some recovery | Cropland - not irrigated to Secondary natural - post 1990 | Restorable |
| 28 | 212 | Lost pre 1990 - some recovery | Erosion to Secondary natural - post 1990 | Restorable |
| 35 | 989956 | Lost pre 1990 - some recovery | Mine to Secondary natural - post 1990 | Restorable |
| 51 | 4153509 | Lost pre 1990 - some recovery | Plantation to Secondary natural - post 1990 | Restorable |
| 59 | 33594261 | Lost pre 1990 - some recovery | Secondary natural - pre 1990 to Secondary natural - pre 1990 | Restorable |
| 27 | 1410127 | Lost pre 1990 - no change | Erosion to Erosion | Restorable |
| 56 | 2 | Lost pre 1990 - no change | Secondary natural - pre 1990 to Erosion | Restorable |
| 1 | 6264538 | Lost pre 1990 - no change | Artificial waterbody to Artificial waterbody | Not restorable |
| 2 | 92774 | Lost post 1990 | Bare ground to Artificial waterbody | Not restorable |
| 9 | 523 | Lost pre 1990 - no change | Built up to Artificial waterbody | Not restorable |
| 16 | 715 | Lost pre 1990 - no change | Cropland - irrigated to Artificial waterbody | Not restorable |
| 18 | 25280 | Lost pre 1990 - no change | Cropland - not irrigated to Artificial waterbody | Not restorable |
| 24 | 125 | Lost pre 1990 - no change | Erosion to Artificial waterbody | Not restorable |
| 29 | 4537 | Lost pre 1990 - no change | Mine to Artificial waterbody | Not restorable |
| 36 | 247255 | Lost post 1990 | Natural to Artificial waterbody | Not restorable |
| 45 | 12716 | Lost pre 1990 - no change | Plantation to Artificial waterbody | Not restorable |
| 52 | 39050 | Lost pre 1990 - no change | Secondary natural - pre 1990 to Artificial waterbody | Not restorable |
| 4 | 47141 | Lost post 1990 | Bare ground to Built up | Not restorable |
| 10 | 25751242 | Lost pre 1990 - no change | Built up to Built up | Not restorable |
| 19 | 290729 | Lost pre 1990 - no change | Cropland - not irrigated to Built up | Not restorable |
| 30 | 13629 | Lost pre 1990 - no change | Mine to Built up | Not restorable |
| 38 | 3607995 | Lost post 1990 | Natural to Built up | Not restorable |
| 46 | 298515 | Lost pre 1990 - no change | Plantation to Built up | Not restorable |
| 53 | 716031 | Lost pre 1990 - no change | Secondary natural - pre 1990 to Built up | Not restorable |
| 65 | 4100 | Lost marine pre 1990 - no change | Built up marine to Built up marine | Not restorable |
| 60 | 10967 | Lost shore pre 1990 - no change | Built up shore to Built up shore | Not restorable |
| 62 | 2388 | Lost shore post 1990 | Natural shore to Built up shore | Not restorable |
| 11 | 78255 | Lost pre 1990 - no change | Built up to Cropland - irrigated | Not restorable |
| 17 | 13734328 | Lost pre 1990 - no change | Cropland - irrigated to Cropland - irrigated | Not restorable |
| 25 | 231 | Lost pre 1990 - no change | Erosion to Cropland - irrigated | Not restorable |
| 31 | 1582 | Lost pre 1990 - no change | Mine to Cropland - irrigated | Not restorable |
| 47 | 54870 | Lost pre 1990 - no change | Plantation to Cropland - irrigated | Not restorable |
| 54 | 390175 | Lost pre 1990 - no change | Secondary natural - pre 1990 to Cropland - irrigated | Not restorable |
| 12 | 1886372 | Lost pre 1990 - no change | Built up to Cropland - not irrigated | Not restorable |
| 20 | 127876567 | Lost pre 1990 - no change | Cropland - not irrigated to Cropland - not irrigated | Not restorable |
| 26 | 20009 | Lost pre 1990 - no change | Erosion to Cropland - not irrigated | Not restorable |
| 32 | 25270 | Lost pre 1990 - no change | Mine to Cropland - not irrigated | Not restorable |
| 48 | 399215 | Lost pre 1990 - no change | Plantation to Cropland - not irrigated | Not restorable |
| 55 | 4738090 | Lost pre 1990 - no change | Secondary natural - pre 1990 to Cropland - not irrigated | Not restorable |
| 13 | 15624 | Lost pre 1990 - no change | Built up to Mine | Not restorable |
| 21 | 397257 | Lost pre 1990 - no change | Cropland - not irrigated to Mine | Not restorable |
| 49 | 39537 | Lost pre 1990 - no change | Plantation to Mine | Not restorable |
| 57 | 126327 | Lost pre 1990 - no change | Secondary natural - pre 1990 to Mine | Not restorable |
| 66 | 94 | Lost marine pre 1990 - no change | Mine to Mine | Not restorable |
| 61 | 1808 | Lost shore pre 1990 - no change | Mine shore to Mine shore | Not restorable |
| 14 | 151068 | Lost pre 1990 - no change | Built up to Plantation | Not restorable |
| 22 | 306109 | Lost pre 1990 - no change | Cropland - not irrigated to Plantation | Not restorable |
| 50 | 16354320 | Lost pre 1990 - no change | Plantation to Plantation | Not restorable |
| 58 | 563389 | Lost pre 1990 - no change | Secondary natural - pre 1990 to Plantation | Not restorable |

## Appendix S6


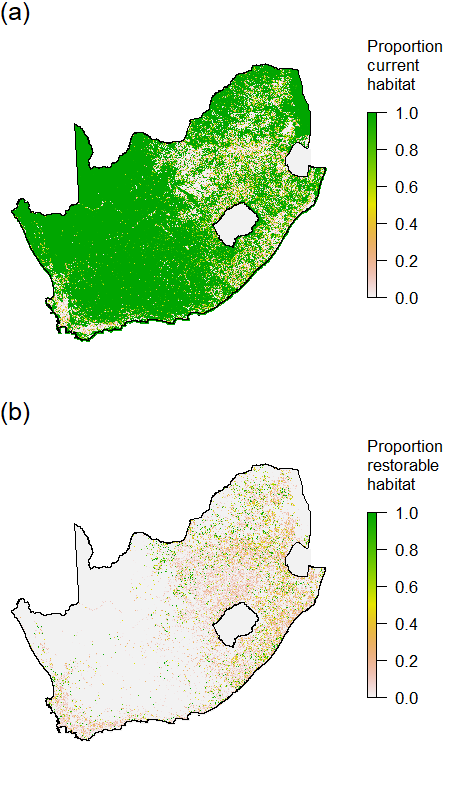


South Africa current natural habitat and restorable habitat maps. **(a)** The proportion of current natural habitat and **(b)** the proportion of restorable habitat, per 300m grid cell in 2014.
